# Neutralization Interfering Antibodies: A “Novel” Example of Humoral Immune Dysfunction Facilitating Viral Escape?

**DOI:** 10.3390/v4091731

**Published:** 2012-09-24

**Authors:** Mancini Nicasio, Giuseppe Sautto, Nicola Clementi, Roberta A. Diotti, Elena Criscuolo, Matteo Castelli, Laura Solforosi, Massimo Clementi, Roberto Burioni

**Affiliations:** Microbiology and Virology Unit, “Vita-Salute” San Raffaele University, via Olgettina 58, Milan 20132, Italy; Email: sautto.giuseppe@hsr.it (G.S.); clementi.nicola@hsr.it (N.C.); diotti.robertaantonia@hsr.it (R.A.D.); e.criscuolo@studenti.unisr.it (E.C.); m.castelli@studenti.unisr.it (M.C.); solforosi.laura@hsr.it (L.S.); clementi.massimo@hsr.it (M.C.); burioni.roberto@hsr.it (R.B.)

**Keywords:** neutralizing antibodies, non-neutralizing antibodies, interfering antibodies, viral escape mechanism

## Abstract

The immune response against some viral pathogens, in particular those causing chronic infections, is often ineffective notwithstanding a robust humoral neutralizing response. Several evasion mechanisms capable of subverting the activity of neutralizing antibodies (nAbs) have been described. Among them, the elicitation of non-neutralizing and interfering Abs has been hypothesized. Recently, this evasion mechanism has acquired an increasing interest given its possible impact on novel nAb-based antiviral therapeutic and prophylactic approaches. In this review, we illustrate the mechanisms of Ab-mediated interference and the viral pathogens described in literature as able to adopt this “novel” evasion strategy.

## 1. Introduction

**Figure 1:**
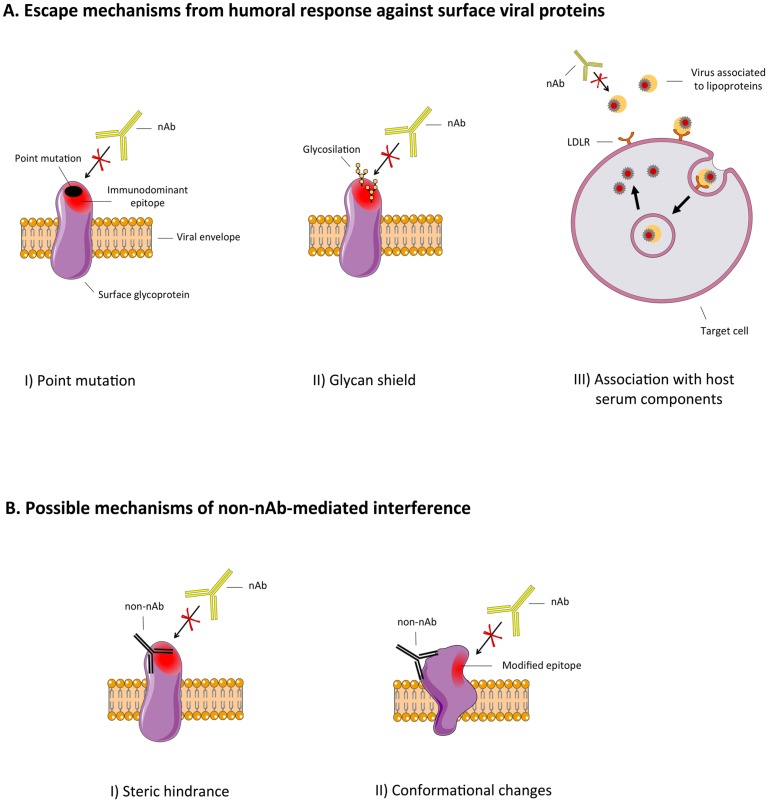
(**A**) Schematic representation of viral escape mechanisms from humoral immune response against surface viral proteins: point mutations on immunodominant regions, glycosylation of functionally pivotal residues (glycan shield) of the viral surface proteins and virus association with host serum components (e.g., lipoproteins) (**B**) Mechanisms of interference on nAb-mediated virus neutralization by the binding of interfering non-nAbs: non-neutralizing/interfering Abs might interfere with the binding of nAbs by steric hindrance following a spatial occupancy of their epitope or a competition for the binding; otherwise the binding of non-neutralizing/interfering Abs may induce conformational changes on the viral protein, thus affecting nAb binding to the antigen. Non‑neutralizing/interfering Abs are depicted in black while nAbs in yellow.

Hypervariable viruses adopt several mechanisms to cope with the host humoral immune response. The most studied mechanism is the accumulation of point mutations on immunodominant regions of surface proteins, making them no longer recognizable by previously generated neutralizing antibodies (nAbs) [1,2,3,4]. Other escape mechanisms involving surface proteins include glycosylation of functionally pivotal residues (the glycan shield) or their association with host serum components (e.g., lipoproteins) in order to mask them from the immune system [5,6,7,8,9] (Figure 1A). Other known escape mechanisms are (i) a sort of protected route of virus spreading, such as cell-to-cell transmission [10,11]; (ii) the molecular mimicry between viral proteins and host self-antigens or (iii) the viral‑induced stimulation of subfamily-restricted antibodies (Abs), both with obvious implications in viral-induced autoimmune diseases such as cryoglobulinemia for HCV [12,13,14]. The possible interfering effect of non-neutralizing Abs (non-nAbs) was originally proposed by Dulbecco *et al.* in 1956 [15], to explain the apparent inhibition of virus neutralization exerted by some serum samples.

Recently, this proposed immune escape mechanism has re-acquired a relevant interest, especially considering the potential clinical use of neutralizing anti-infectious nAbs or the design of epitope‑based vaccinal approaches [16]. To date, two main mechanisms have been proposed for the interfering effects of non-nAbs: (i) direct binding interference by steric hindrance, (ii) inhibition of binding following conformational changes of the viral antigen bound by interfering non-nAbs.Moreover, it has been speculated that, even when not directly interfering with nAbs binding, non-nAbs may also lead to the enhancement of viral infection through interaction with Fc receptors or complement receptors [17]. 

Overall, possibly elicited non-nAbs in infected or vaccinated individuals may interfere with the neutralizing potential of nAbs. In more detail, these interfering Abs are able to bind viral proteins at the level of immunodominant but functionally irrelevant regions of viral proteins, decreasing or blocking the binding of nAbs to crucial viral epitopes (e.g., receptor-binding domains) (Figure 1B) [18]. A candidate antiviral monoclonal antibody (mAb) or polyclonal preparation should not be subjected to this mechanism of interference, or to the other escape mechanisms previously mentioned. Similarly, novel vaccinal approaches should avoid the elicitation of interfering Abs that could even worsen the disease in case of a real infection. 

In the following paragraphs we discuss these mechanisms with specific examples of their role in the course of the viral infections where they have been described.

## 2. Hepatitis C Virus (HCV)

Hepatitis C virus (HCV) is a positive-sense single stranded RNA enveloped virus causing chronic hepatitis in most untreated patients (about 80%), with the consequent risk of developing cirrhosis and hepatocellular carcinoma. More than 170 million people (2%–3% of the world population) are infected worldwide, and a protective vaccine is not yet available, whereas therapeutic options are still limited and not completely effective [19]. For these reasons chronic HCV infection represents the major indication for liver transplantation in Europe and United States. Moreover, transplanted recipients are subject to high risk of graft re-infection and to a more severe and rapid progression of the liver disease [20]. 

The HCV genome encodes a single polyprotein of about 3,000 aminoacids that is processed by host and viral proteases into at least 3 structural (core, E1 and E2) and 7 non-structural (p7, NS2, NS3, NS4A, NS4B, NS5A and NS5B) proteins [21,22]. In particular, the envelope type I membrane glycoproteins E1 and E2 form non-covalent heterodimers on the surface of the HCV envelope and allow clathrin-mediated virus endocytosis interacting consecutively with several entry cellular factors such as glycosaminoglycans [23,24,25], low-density lipoprotein receptor [26,27], scavenger receptor class B type I [28], the tetraspanin CD81 [29], the tight-junction proteins claudin-1 and occludin, and the recently described Niemann-Pick C1-like 1 cholesterol absorption receptor [30,31,32,33,34]. The development of effective prophylactic and therapeutic approaches against this virus has been hindered mainly by its high mutation rate that gives rise to highly diversified viral variants, even within a single patient (quasispecies) [35]. Indeed, seven major genotypes, varying by up to 30% in nucleotide sequence, and several subtypes are recognized, each characterized by different clinical features such as different evolutionary rates to chronic liver diseases or different response to available antiviral therapies [21,36,37]. 

The development and use of anti-HCV mAbs capable of targeting structurally and functionally conserved regions of the highly variable viral particles are being considered as novel therapeutic tools [38,39,40,41,42,43]. In particular, the production of potent nAbs in acute infections has been shown to correlate with viral clearance in a single-source outbreak cohort [44]. Moreover, in vaccinated chimpanzees, a sustained Ab response to envelope glycoproteins E1 and E2 correlates with reduced viremia [45], while the passive administration of neutralizing mAbs in a uPA-SCID chimeric mouse model of infection was able to protect against challenge with a HCV quasispecies inoculum [46]. Broadly cross‑neutralizing human mAbs directed against the surface E2 glycoprotein of HCV (HCV/E2) are typically directed against functionally important regions within the CD81 binding site [47,48,49,50,51,52,53,54], as well as against other critical residues highly conserved among different genotypes [55,56]. This aspect is crucial for the possible therapeutic *in vivo* use of such mAbs, but it may not be sufficient since it has been recently supposed that other non-nAb populations may interfere with their neutralizing activity [39,57,58,59,60,61]. In fact, in persistently infected individuals anti-HCV/E2 cross-nAbs are generally elicited at low titer and in a late stage of the infection, leading to a poor control of viremia, whereas quasispecies-specific neutralizing or high titer non-nAbs are elicited earlier [53,58,59,60,61,62]. Moreover, the *in vivo* use of anti-HCV polyclonal immunoglobulin preparations in both chimpanzees and humans has been disappointing, and clinical studies have shown that these preparations fail to prevent recurrent infections in patients after liver transplantation [63].

At this regard, a recent paper has suggested that the effect of some of these nAbs, directed against functionally important residues involved in the viral binding to CD81 (within epitope I, encompassing aminoacid residues 412–426), could be hindered by the presence of non-nAbs binding residues within epitope II on HCV/E2 (aminoacid residues 434–446) [58]. In particular, blocking of these interfering epitope II-specific Abs not only raised the neutralizing titer of serum containing both epitope I- and epitope II-specific Abs, but also uncovered a broader cross-genotype neutralizing response [58].

However, the role (and the existence itself) of these interfering Abs in influencing HCV infection is still controversial. Some authors recently corroborated the data of Zhang *et al.* by *in vitro* neutralization assays using serum-derived HCV of genotype 4a and polyclonal Abs derived from immunized goats with different conserved peptides spanning aminoacid residues 412–419, 430–447 and 517–531 of HCV/E2 glycoprotein [64]. In particular, this group found an interfering activity exerted by the weakly neutralizing 430–447-elicited Abs on the neutralizing activity of both the 412–419 and the 517–531-elicited Abs [64]. Interestingly, according to the putative model for E2 folding, all the three aforementioned regions would lie next to each other on the glycoprotein [48]. Therefore, this structural prediction possibly supports the interfering effect of epitope II-directed Abs. However, while this predicted structure is currently the best model available, these conclusions cannot be absolutely ascertained. For this purpose, the availability of E1-E2 crystal will certainly accelerate the fine elucidation of the spatial proximities of neutralizing and interfering mAbs on the E1-E2 structure and, consequently, structure-based vaccine progress.

Moreover, it is noteworthy that individuals with Abs that target the region of E2 encompassing epitope I frequently harbor Abs that recognize the region containing epitope II, thus confirming the co‑immunogenicity of these epitopes [58]. Finally, it has been shown both a low prevalence (less than 2.5%) and a low titer of epitope I-reactive Abs in sera from both chronic and acute resolved infections thus supporting the hypothesis of a conformational masking by adjacent regions such as that containing epitope II [65]. In fact, Zhang *et al.* originally put forward the idea that once epitope II is bound to an Ab, the site of epitope I becomes masked and can no longer be recognized by specific nAbs. Indeed, depletion of Abs to epitope II in plasma from a chronically infected HCV patient and vaccinated chimpanzees recovered an otherwise undetectable cross-genotype neutralizing activity [58]. Another possibility is that the initial binding of interfering Abs to the region containing epitope II may induce conformational changes on E2 that inhibit the binding by epitope I-directed Abs, as recently suggested by Lapierre *et al.* for other anti-HCV/E2 Abs [66]. 

Conversely, these conclusions were not supported in a recent study by Tarr *et al.* using murine (AP33) and rat (2/69a) mAbs, as well as human immunoglobulin fractions affinity-purified on linear peptides representing distinct HCV/E2 domains clustering within the regions 412–426 and 434–446 [67]. Although confirming the previously reported co-immunogenicity of these two regions, the authors failed to demonstrate any inhibition between these two groups of Abs. Considering their results, the authors indeed suggested that interference by non-nAbs, at least to the region encompassing residues 434–446, is not a possible mechanism for HCV persistence in chronically infected individuals, as it had been originally proposed by Zhang *et al.* In accordance with the findings of Tarr and colleagues, Keck *et al.* described anti-HCV/E2 human mAbs binding conformation-sensitive epitopes encompassing also some residues within the 434–446 interfering region [56]. These mAbs are broadly neutralizing and do not lead to viral escape mutants, demonstrating the functional importance of their epitopes. The authors conclude that not all Abs directed against epitope II are interfering, but they also speculate that the interfering activity could be limited to Abs recognizing linear epitopes within it [56]. 

Recently, we have partly confirmed the observations of Zhang *et al.* using a panel of anti-HCV/E2 mAbs: the well characterized mouse anti-HCV/E2 mAb AP33, whose epitope encompasses epitope I (aminoacid residues 412–423), and a weakly neutralizing human anti-HCV/E2 mAb (named e509), whose epitope encompasses epitope II [68]. In particular, we found that e509 is able to interfere with the neutralizing activity of AP33 on genotype 1a virus (strain H77). Instead, we found that e509 does not minimally interfere with the activity of two other broadly cross-neutralizing human anti-HCV/E2 mAbs, named e20 and e137 [49,69]. Interestingly, we found that both e20 and e137 bind also residues within epitope II, at a higher affinity compared to e509, thus displacing it from the interfering epitope and, therefore, keeping unaltered their neutralizing activity. Thus, in our opinion, the described divergent observations reported above may depend on the different Ab specificities present in the polyclonal preparations used and, probably, also on the different HCV genotypes infecting the studied patients [68]. Moreover, the different strategies adopted in isolating epitope I- and epitope II-directed Abs followed in the studies above could explain the different data obtained. In fact, immunoglobulins purified on peptides representing distinct HCV/E2 regions [67] are obviously directed against linear epitopes; these preparations are certainly different from mAbs cloned using a full-length HCV/E2 glycoprotein, which are more probably directed against conformational epitopes including also residues outside the investigated linear regions [54]. 

To summarize, in the HCV field several works support the existence of interfering Ab populations and hypothesize their possible role in HCV persistence, as demonstrated using human plasma-derived immunoglobulin preparations, human mAbs, and sera of animals vaccinated with recombinant HCV/E2 peptides. The possible mechanism leading to the interference is still controversial, but both direct steric hindrance and induced antigen conformational changes have been hypothesized. On the other hand, other papers do not confirm these findings, suggesting that the putative interfering epitope II may be targeted by Abs endowed with a broadly neutralizing activity. Our recent paper, using well characterized mAbs [68], shows that the interfering Abs do exist but that their overall effect may be biased by the presence of nAbs with different binding features and by the infecting HCV genotype. Future works investigating the *in vivo *role of these interfering Ab subpopulations in HCV persistence will certainly be very useful.

## 3. Influenza Viruses

The influenza viruses circulate worldwide in animal reservoirs, especially water fowl, potentially affecting humans of any age group. Influenza viruses are classified into types A, B or C based on antigenic differences of their nucleoprotein and matrix protein. The most clinically relevant and variable type is influenza A which is divided in several subtypes, according to the antigenic characteristic of the two envelope glycoproteins, and causes epidemic and pandemic infections [70]. The yearly recurring influenza epidemics are associated with significant morbidity and mortality, particularly among risk groups (such as elderly people or those with chronic medical conditions, pregnant women and children) [71]; the global spread of pandemic influenza viruses can cause millions of deaths [72]. 

Within the enveloped influenza virion eight segments of negative single-stranded RNA are protected by the nucleocapsid protein, forming the ribonucleoprotein (RNP). The first six RNA segments each code for a single protein: PB2, PB1, and PA (all constituting the RNA-dependent RNA polymerase), the hemagglutinin (HA), the nucleoprotein (NP), the neuraminidase (NA). The last two segments each code for two different proteins: the matrix proteins (M1 and M2) and the non-structural proteins (NS1 and NS2). Three different proteins (HA, NA and M2) are present on the viral envelope. The HA glycoprotein is the most abundant and it is the major target of the humoral immune response. Together with the NA transmembrane glycoprotein, HA is capable of eliciting a subtype-specific immune responses which is fully protective within, but only partially protective across different subtypes [73]. HA is synthesized as inactive precursor that transits into its active form upon cleavage by host cell proteases, and which is present on the viral membrane as homotrimers. HA trimers bind to 2,6-linked sialic acid molecules on cell membrane proteins or lipids through domains located in the globular head of each monomer. Subsequently, the viral envelope fuses by clathrin-dependent and -independent mechanisms with the endocytic vesicle membrane through the HA fusion peptide located in the stem region of each monomer. As a consequence, viral components are released into the host cell and can subvert the synthetic capabilities of the host cell for production and release of progeny particles [74].

The humoral immunity plays an important role in the host defense against influenza virus infection as most of Abs neutralize influenza viruses and, hence, limit infection [75,76,77,78]. In fact, a large body of experimental works suggests that occlusion of the receptor-binding site on HA by Abs is the main mechanism of influenza viral neutralization. Less common, but more broadly nAbs may neutralize influenza virus by inhibiting fusion of the viral envelope with the endocytic-vesicle membrane [50,79,80,81,82,83]. Aminoacid changes on HA, more frequent on the immunodominant globular head, have complex effects on viral neutralization by Abs, usually allowing the mutated variants to escape from previously generated nAbs [84]. Classical studies using neutralizing mouse mAbs identified five distinct antigenic sites (A–E) on the HA1 globular head region in the three-dimensional structure of the H3 HA molecule (A/Hong Kong/1/68) [85,86,87] as well as in H1 [88] and H2 subtypes [89].

During the first few days of an infection, the nAb titer is often low, while the titer of non-nAbs is higher and may play a role in the outcome of an infection, as recently observed for influenza A/2009 H1N1 pandemic virus infected patients by To *et al.* [90]. In particular, this group found that the amount, as well as the avidity, of non-nAbs were higher for patients with severe disease than for those with mild disease. The authors concluded that an exaggerated non-nAb response during the early stage of infection was associated with severe disease [90]. Moreover, the authors speculated that non-nAbs present in patients’ sera during the early stage of infection were likely to be either preexisting or the result of a secondary heterosubtypic humoral immune response against more conserved epitopes on several influenza proteins [91]. This early humoral response can be elicited within a few days after infection, because of immune priming by previous exposure to shared viral epitopes. In fact, the matrix proteins and nucleoprotein have conserved aminoacid sequences, and therefore Abs against these proteins from previous seasonal influenza virus infection or vaccination could be induced [92]. Indeed, upon infection with influenza virus, memory B-cells can proliferate rapidly and generate a large amount of these high avidity non-nAbs, especially in patients with severe disease. This is consistent with the observation that the number of peripheral blood B-cells is higher in patients with severe disease than in those with mild disease during the early stage of infection.

The mechanism of Ab neutralization interference has been indirectly speculated also by Ndifon *et al.*, who observed that some aminoacid changes on HA actually increase the efficiency of neutralization of escape variants by previously generated Abs, even if not directly influencing their binding [93]. In detail, this group suggested that the increase in neutralizing activity after HA mutation could be the resultant of a lesser steric interference between Abs. Specifically, if there is a steric competition for binding to HA by Abs with different neutralization efficiency, then a mutation that reduces the binding of Abs with low neutralizing activity could increase the overall viral neutralization. Indeed, similarly to what has been speculated for HCV, Abs that bind to HA epitopes located at a distance from the receptor-binding site may therefore fail to occupy this site efficiently, thereby leading to a decreased viral neutralization. Moreover, it has been shown that Abs that bind to a certain HA epitope can prevent further binding of Abs to other epitopes of the same HA protein, and even to epitopes found on adjacent HA proteins. The above observations suggest that Abs that bind to low-neutralization efficiency epitopes of HA might interfere with the binding of nAbs to close high‑neutralization efficiency epitopes, thereby impeding the neutralization of influenza viruses. Considering the HA structure, the binding of the interfering Abs would lie at the level of epitope C and E located far from the receptor-binding site on the globular head of the HA. However, the binding of these Abs may influence the binding of nAbs to epitopes A, B and D, located closer to the receptor-binding site [93]. At this regard changes to epitope A, B and D could be highly favored by natural selection, whereas changes to epitopes C and E could be disadvantageous to influenza viruses [93].

Similarly to HCV, but with a sounder confirm due to the availability of the crystal structure, these speculations raise the intriguing possibility that the influenza viruses may have evolved by favoring the preferential elicitation of Abs recognizing epitopes with a low-neutralization profile. Indeed, steric hindrance by Abs that bind these epitopes could greatly reduce the extent of mutation required for a virus to evade neutralization by host Abs. Consequently, a decrease in the affinity of Abs for epitopes with low-neutralization efficiency could lead to an increase in viral neutralization. This suggests a possible approach to design “low-interference” vaccines that could greatly diminish the impact of Ab interference. These immunogens are genetically modified from viral target only at the level of low‑neutralization efficiency epitopes. Indeed, vaccine-induced Abs only recognize high-neutralization efficiency epitopes of the target and Abs induced by low-interference vaccine strain have low affinity for low-neutralization efficiency epitopes of the target circulating virus strain. Therefore, they do not interfere with Abs to high-neutralization efficiency epitopes, implying an improved neutralization. Consequently, limiting Ab-mediated interference, the target virus cannot escape from vaccine-induced Abs through small epitope changes. Alternatively, vaccines could be designed to include only those regions that correspond to epitopes with high-neutralization efficiency.

Furthermore, antiviral drugs could be designed to include viral proteins carrying modifications at the level of high-neutralization efficiency epitopes; these “decoy” proteins would compete with virus for binding to low-neutralization efficiency Abs in a manner similar to that played by neuraminidase inhibitors.

In synthesis, the availability of HA crystal structure has helped to confirm the existence and to explain the mechanisms of interference by non- or weakly-neutralizing anti-HA Abs. The recent work by To *et al.* [90] evidencing that a non-nAb response during the early stage of infection is associated with a severe disease, may be the first proof of the role of these interfering Abs in the course of a natural infection.

## 4. SARS Coronavirus (SARS-CoV)

The Severe Acute Respiratory Syndrome Coronavirus (SARS-CoV) is a positive and single-stranded RNA virus emerged in 2002 in Guangdong, People’s Republic of China, and spread to 26 countries in 2003. Infection control efforts brought the infection under control by mid-2003 [94]. More than 8,000 cases, including almost 800 deaths, were reported during the outbreak period and increasing age and comorbidity were risk factors for severe disease and death [95]. Since 2003, only sporadic cases have been reported; however, the possibility that SARS outbreaks could reemerge naturally or be deliberately released is a public health concern. Like influenza viruses, SARS-CoV circulates in animal reservoirs, with bats that are thought to transmit the virus to small mammals with exposure to these small animals as the source of human infections [96]. The clinical disease is similar to other severe acute respiratory infections, including influenza, and the SARS case definition includes clinical, epidemiologic, and laboratory criteria [97,98].

The basic genome organization and replicative cycle is similar for all CoVs*.* Gene 1 encodes all predicted replicase/transcriptase proteins, which are translated from input genomic RNA, while genes 2–9 encode structural and accessory proteins, including the envelope spike (S) protein, which are translated from separate subgenomic mRNAs. CoVs use a unique discontinuous mechanism to transcribe a series of progressively larger subgenomic mRNAs, and each contains a leader RNA sequence that is derived from the 5' end of the genome [99].

The S protein of CoVs is inserted in the envelope of the virion mediating binding and fusion events necessary for infection, and it is the major target of the humoral protective immunity [100]. Although the S protein of SARS-CoV (SARS-S) shares little aminoacid identity (approximately 20%–27%), it shares common structural features with S proteins of the other members of the *Coronaviridae *family. SARS-S protein is a type I transmembrane glycoprotein of approximately 1,255 amino acids in length and divided into two functional domains: S1 (aminoacid residues 15–680) and S2 (aminoacid residues 681–1,255) [101]. In many CoVs, the S protein is cleaved during biogenesis and these two functional domains are held together non-covalently; however, as in the case of human CoV 229E, the S protein is not cleaved in SARS-CoV [102]. The S1 domain forms a globular structure that mediates interaction of the S protein with its main receptor, angiotensin-converting enzyme 2 (ACE2), while the S2 domain mediates fusion and contains the putative fusion peptide and two conserved helical regions (HR1 and HR2) that upon cleavage by the endosomal protease cathepsin L form the six helix bundle fusion core [103]. 

Vaccine strategies aiming at blocking/limiting infection by SARS-CoV mainly focus on targeting the SARS-S viral glycoprotein [100]. Nonetheless, such a strategy poses a singular dilemma for CoVs, as previous vaccination protocols have highlighted the possibility of immune-mediated enhancement of the disease [104].

At this regard, the group of Zhong *et al.* investigated the role of non-neutralizing interfering Abs also in the case of SARS-CoV infection [105]. In particular, they found that two mAbs directed against the region encompassing aminoacid residues 491–510 of SARS-S (341C and 540C) act synergistically to inhibit SARS-CoV infection *in vitro*, while a non-neutralizing mAb (240C) whose epitope encompasses the above mentioned region, disrupted the neutralizing activity of both 341C and 540C [105,106]. By analyzing the crystal structure of the SARS-S protein, the authors proposed a possible explanation to what observed, evidencing that the epitopes of all the mAbs are closely packed and proximal to each other but distal from the ACE2 receptor binding site [105]. Moreover, the epitope of the non-neutralizing mAb 240C partially overlaps by at least 2 aminoacids (P507 and A508) with that of the neutralizing mAb 341C. As a consequence, mAb 240C could inhibit mAb 341C binding in an equilibrium-related manner. On the other hand, the authors found that the 240C mAb could sterically interfere with the binding of the 540C mAb through the proposed mechanism of spatial occupancy (Figure 1B). In fact, the accessibility of mAb 540C to its epitope may be blocked by the mAb 240C binding that masks the surface area containing it. In fact, as speculated by Davies and Cohen, the buried area of an Ab can range from 500 Å^2^ to more than 800 Å^2^ corresponding to 21–32 aminoacids, although only 9–20 aminoacid residues (the real epitope) make direct contacts with the Ab [107]. In fact, as previously observed for HCV, influenza and other human and animal viruses [108], one of the possible mechanisms is that the steric block by non-nAbs reduces the binding of nAbs on the SARS-S protein disabling neutralization. Conversely, notwithstanding the epitopes of mAbs 341C and 540C are located on a single loop; they are spatially separated thereby providing distinct interfaces for independent Ab binding.

To conclude, SARS-CoV can elicit potentially interfering non-nAbs by presenting on its surface closely packed regions with different biological features. On the other hand, the host can mount a vigorous neutralizing humoral response by producing Abs that recognize distinct epitopes and act synergistically. In particular, these results suggest that a cocktail of neutralizing human mAb that can bind to unique epitopes and have different mechanisms of action might be of clinical utility against SARS-CoV infection, and indicate that a similar approach may be applied to treat other viral infections [109].

## 5. Human Immunodeficiency Virus (HIV)

The human immunodeficiency virus (HIV) is a positive single-stranded RNA retrovirus, causing substantial morbidity and mortality across the globe, particularly in developing countries. Human immunodeficiency viruses type 1 and 2 (HIV-1 and HIV-2) are the results of multi-interspecies transmissions from simian virus to humans. HIV-2 prevalence is low and there is an higher proportion of HIV-2 infected individuals that do not progress to acquired immunodeficiency disease syndrome (AIDS) compared with those infected with HIV-1 [110]. HIV-1 viruses are very divergent and are classified in four groups: M, N, O and P. In particular, the group M is subdivided in nine subtypes and numerous circulating recombinant forms [111].

The genome of all retroviruses encode the Gag, Pol and Env structural proteins. Among the HIV structural proteins, gp120 and gp41 surface envelope glycoproteins form heterodimers that are organized as trimers on the surface of the viral membrane. HIV-1 entry into target cells is initiated by the interaction of these surface envelope glycoproteins with CD4 and a co-receptor (typically CCR5 or CXCR4) on target cells [112]. The gp120 portion binds the target cell receptors, while gp41 promotes fusion of viral and cellular membranes [113]. Upon binding to the CD4 receptor, gp120 undergoes a conformational change, resulting in the exposure of epitopes that can be bound by co-receptor molecules and in the eventual formation of the transient pre-hairpin intermediate conformation [114,115,116]. In the pre-hairpin intermediate, the gp41 molecules reorganize so that the N-terminal peptides form a trimer of helices that expose the fusion peptide to the target cell, while the C-terminal helices remain anchored to the viral membrane [113]. This stage is vulnerable to a number of nAbs and peptides capable of binding either the N- or C-terminal peptides [117,118]. Upon fusion with the target cell membrane, further gp41 reorganization results in the association of N- and C-terminal peptides to create a six-helix post-fusion bundle [119]. After fusion and delivery of the viral capsid in the cytoplasm, uncoating leads to the release of viral enzymes, proteins, and genomic RNA inside the cell. Reverse transcription of the viral genomic single-stranded positive RNA is then initiated to yield a double-stranded proviral DNA to be imported in the nucleus and integrated into host chromosome. Active transcription from the integrated proviral DNA occurs in the presence of NF-κB and viral Tat. Splicing of viral mRNA yields early accessory proteins like Tat, Rev, and Nef, which help in transcription, splicing, and modification of the cellular machinery, respectively. Accumulation of Rev protects the viral mRNA from splicing, thus yielding increasingly longer mRNAs able to code for structural and envelope proteins, and finally viral genomic RNAs is ready to be encapsidated [111].

Antiretroviral drug therapy for HIV is highly effective in controlling the infection; however, the eradication of this virus is currently not practicable and the treatment is therefore lifelong and burdened by considerable toxicity and drug resistance. A vaccine is widely viewed as being crucial for the control of the epidemic but several advanced efforts to develop an effective prophylaxis resulted unsuccessful [120,121]. One of the greatest challenges in developing a vaccine against HIV is to overcome its ability to constantly mutate and escape anti-HIV immune responses [122]. This high mutation rate is a direct result of the presence of the virus’ low fidelity RNA polymerase as well as the high levels of recombination it undergoes and the constantly evolving glycan shield of the envelope glycoproteins [123,124,125]. At this regard, both cytotoxic T lymphocytes and nAbs have long been reported to select for immune escape variants during the course of HIV-1 infection [126,127,128].

A candidate passive immunotherapy could consist, as previously suggested for SARS-CoV infection, in the administration of a cocktail of broadly neutralizing mAbs, that could minimize the onset of viral escape mutants [129]. Various combinations of human mAbs have been studied over the past several years which have shown additive, synergistic, or antagonistic effects on the neutralization of HIV-1 [130,131,132,133,134,135]. Antagonistic effect in HIV-1 neutralization has been previously reported with a pair of anti-gp120 mAbs directed against the V3-loop and the CD4 binding site, respectively [136]. The molecular mechanisms determining the antagonism have not been further studied in details.

The only study describing for the first time at the molecular level a possible mechanism of interference also for HIV was performed using pair combinations of anti-gp41 mAbs [137]. More in details, the authors noted an antagonistic effect when the anti-gp41 neutralizing mAbs 2F5 or 50–69 were combined with the non-neutralizing anti-gp41 mAb 98–6 [137]. In particular mAbs 50–69 and 98–6 recognize different gp41 epitopes located within cluster I (aminoacid residues 579–613) and cluster II (aminoacid residues 644–667), respectively. On the other hand mAb 2F5 recognize a different epitope from mAb 98–6, within the gp41 membrane-proximal external region (MPER), in a portion adjacent to the cluster II region of gp41. Moreover, there is some overlap between cluster II epitopes and the epitope recognized by mAb 2F5 [138], explaining the inhibition of mAb 2F5 binding by mAb 98–6 [137,139].

Thus, in the case of the antagonism between mAbs 2F5 and 98–6 the author hypothesized a mechanism of steric hindrance between the two mAbs as they could bind peptides and peptide complexes representing the pre-fusogenic and fusogenic forms of gp41 [140]. In particular, mAb 98–6 had a higher affinity for the peptide complexes representing the fusogenic form, than did 2F5. Thus, the binding of 98–6, which fails to neutralize the HIV-1 isolate 89.6 (HIV-1_89.6_), could interfere with the binding of 2F5, leading to the neutralization antagonism. In contrast, mAbs 50–69 and 2F5 recognize distinct epitopes on gp41, and display independent (additive) reactivity against HIV-1_89.6_ in combination with most of the other anti-gp41 and anti-gp120 mAbs tested [137]. 

To conclude, anti-gp120 and anti-gp41 Abs are induced in HIV-1-infected individuals but are predominantly non-neutralizing, since the functionally important regions of HIV surface proteins are almost completely hidden to the immune system [139]. An intriguing hypothesis is that, together with other HIV escape mechanisms, the effect of the extremely rare anti-gp41 and anti-gp120 nAbs may be also hindered by the overwhelming amount of interfering non-nAbs. To date, the existence of interfering non-nAbs has been clearly evidenced only using anti-gp41 mAbs with different biological features, whereas no data have been generated using anti-gp120 mAbs. The possible role of non-nAb-mediated interference in facilitating HIV escape in the course of the natural infection certainly deserves future studies. 

## 6. Conclusions

Immunoprophylactic or immunotherapeutic approaches with mAbs are still considered a possible supporting tool in the management of infectious diseases. In particular, the availability of broadly neutralizing mAbs directed against viral pathogens, whose actual prophylactic and therapeutic approaches are far from effective, has led to many ongoing clinical trials. However, the evidence reported in this review suggest that candidate mAbs to be possibly used in antiviral passive immunization approaches, or to be elicited by future vaccine strategies, have not only to be highly cross-neutralizing molecules [141,142], but also tailored molecules whose activity is not influenced by possible interfering Abs produced in the course of infection. To this end, they must either be directed against highly neutralizing epitopes not subjected to the mechanism of interference, or must feature high affinity for the antigen in order to displace the binding of possible interfering Abs [51,68]. 
